# Why it is good to communicate the bad: understanding the influence of message framing in persuasive communication on consumer decision-making processes

**DOI:** 10.3389/fnhum.2023.1085810

**Published:** 2023-09-05

**Authors:** Nadine R. Gier, Caspar Krampe, Peter Kenning

**Affiliations:** ^1^Faculty of Business Administration and Economics, Heinrich Heine University Düsseldorf, Düsseldorf, Germany; ^2^Marketing and Consumer Behaviour Group, Wageningen University and Research, Wageningen, Netherlands

**Keywords:** message framing, decision-making, fMRI, consumer neuroscience, brain network perspective, persuasive communication, sustainable marketing, reflective-impulsive model

## Abstract

**Introduction:**

One approach to bridging the gap between consumer intentions and behavior is persuasive communication to reinforce their intentions and thereby support their behavior change. Message framing has proven to be a useful, persuasive communication tool. However, message framing is considered more complicated than other types of framing because, in addition to concept-specific elements, it is also strongly influenced by and, in turn, influences emotions. Therefore, it is almost impossible for consumers to verbally express their attitudes, so the challenge is to explain and measure its impact. This research aims to help in this regard by suggesting a theoretical model to understand how message framing is processed from a consumer neuroscience perspective. More precisely, the factors that constitute message framing are systematized and built on a reflective-impulsive model and a neural emotion-cognition framework interpreted to explain the persuasive effects of message framing.

**Method:**

A functional magnetic resonance imaging (fMRI) experiment is used to examine the effects of message framing for four different frame types that are hypothesized to affect consumer information processing differently.

**Result:**

The results suggest that communication strategies should take into account the valence of the objects and the frame used. The behavioral results partially confirm the assumption that two types of information processing could take place, as suggested by the reflective-impulsive model. At the neural level, using the network perspective, the results show that certain brain regions primarily associated with emotional and cognitive interaction processes are active during processing, depending on the framing of the message.

**Discussion:**

In cases of indirect avoidance value-consistent framing, it may be good to communicate the bad in the appropriate frame to influence information processing.

## 1. Introduction

Message framing (or goal framing), meaning to present information that promotes a particular action or behavior by describing the consequences of acting or not acting as a gain or loss, has proven to be a useful persuasive communication tool (Gallagher and Updegraff, 2012; Ainiwaer et al., 2021; Florence et al., 2022). Especially when it comes to sustainability issues, such as sustainable food consumption, where sustainable production may be achieved only through consumer behavior change (Padel and Foster, 2005; Vermeir and Verbeke, 2006; Vanhonacker and Verbeke, 2014), message framing has been examined (Florence et al., 2022), aiming to provide better information to consumers and to encourage more sustainable purchasing decisions (Frank and Brock, 2018; Bundesministerium für wirtschaftliche Zusammenarbeit und Entwicklung, 2022). The intended goal is, thus, to strengthen intentions through persuasive arguments to effectively change behavior at the point of purchase. This is a goal that has yet not fulfilled, requesting to think above and beyond labeling approaches (Gier et al., 2018; Ingenbleek and Krampe, 2022).

Message framing is considered more complicated than other framing types (e.g., attribute or risky choice framing) because “more than one aspect of the message can be manipulated” (Levin et al., 1998, p. 173). This increases the susceptibility of message framing effects to different linguistic (e.g., use of negations) and contextual variations (Levin et al., 1998). In addition to these concept-specific elements, previous review articles and meta-analyses on message framing in marketing, medicine, and communication research (Gallagher and Updegraff, 2012; Nabi et al., 2020; Xu and Huang, 2020; Ainiwaer et al., 2021; Florence et al., 2022) suggest that message framing highly depends on and is, in turn, influenced by a variety of other factors, such as the subjective emotional state and the resulting differences in emotional attitudes. This makes it almost impossible for consumers to verbally express their attitudes as they are constantly influenced and adjusted throughout the process (Paulhus, 1989; Razavi, 2001). Marketing managers and policymakers are therefore advised to carefully define factors for their communication strategies and tools to predict how they might influence people's behavior. Moreover, the challenge remains to measure the effects of message framing because emotions seem to depend on frames and frames are in turn influenced by emotions (Nabi et al., 2020). Accordingly, the question arises of how to explain and measure the effects of more complex message framing that involves multiplicative inverse emotions of frames and objects.

In general, multiple theories have been suggested to explain message framing. A meta-analysis of environmental message framing identified 30 different theories that had been applied in message framing studies (Florence et al., 2022). In addition to the most popular theories applied to develop frames, for example, construal level theory (Trobe and Liberman, 2010) or prospect theory (Tversky and Kahneman, 2018), other theories have been applied to try to explain how message framing impacts consumer behavior. In this context, some studies indicate that a dual-process model might explain the interaction of emotions and message framing (Baek and Yoon, 2017). However, since emotions appear to be influenced in both directions by frames and objects, it is difficult to distinguish the cause and effect of emotional responses to message framing, especially when using self-reports (Bieg et al., 2014).

This is where this study comes in and attempts to extend the explanation of message framing to include and measure the underlying processing mechanisms that constitute this effect. To achieve a better understanding of unconscious and emotionally influenced processes, insights from consumer neuroscience have been proven to enrich theories, providing a way to directly assess processing mechanisms in the individual (Yoon et al., 2012; Smidts et al., 2014; Plassmann and Karmarkar, 2015; Karmarkar and Yoon, 2016). Consequently, a first attempt is made to suggest a model that incorporates neuroscientific evidence into its theoretical considerations (Smith and DeCoster, 2000; Lieberman et al., 2002; Strack and Deutsch, 2004) and to investigate its assumed emotionally influenced processing mechanisms with the help of neuroscientific methods. Moreover, a dual-process approach is taken, which appears to provide a framework for explaining decision-making in a variety of domains, integrating other theories that try to explain persuasion effects, such as the regulatory focus theory (Higgins, 1997; Strack and Deutsch, 2004). The reflective-impulsive model addressed here is among the more recent publications about well-known dual-process theories (Epstein, 1994; Smith and DeCoster, 2000; Lieberman et al., 2002; Kahneman, 2003) and is most cited among neuroscience-based approaches (Loewenstein and O'Donoghue, 2004; Benhabib and Bisin, 2005; Botvinick and Cohen, 2014), being regularly listed in literature review articles (Gawronski and Creighton, 2013; Grayot, 2020; Perugini et al., 2021) and still being cited in recent publications (Exelmans and Van den Bulck, 2021; Li et al., 2021; Schoor and Schütz, 2021; Nowlan et al., 2022).

This research aims to understand the processing of message framing at a consumer level and to identify the effects on consumer behavior from a more fundamental, neuroscientific perspective. Building on the reflective-impulsive model and a neural emotion-cognition framework, the impact of message framing is investigated using functional magnetic resonance imaging (fMRI) for four different frame types that are assumed to impact consumer information processing differently. By doing so, this study attempts to systematize the factors that constitute message framing and contributes to an integrative theory of message framing by using a neuroscientific information processing model as a theoretical basis.

The remainder of this article is structured as follows: In the theoretical background section, first a literature review of message framing and its impact on and influence by emotions is provided. Next, the reflective-impulsive model is introduced as a theoretical foundation that is extended into neural correlates in the light of a network perspective of emotion-cognition interaction, focusing on relevant features for the hypothesis derivation. Subsequently, the applied behavioral and neuroscientific mixed-method is explained before data collection and analysis are presented. In the results section, both behavioral and neural results are presented. This study concludes with a discussion of the results and theoretical contributions, indicating possible implications for research and practice.

## 2. Theoretical background

### 2.1. Message framing: definition and effects

Message framing or goal framing, as a tool of persuasion, has been an important research topic during recent decades (Levin and Gaeth, 1988; Levin et al., 1998; Nabi et al., 2020; Florence et al., 2022). Message framing addresses the consequences of an action or behavior as gain or loss when it is (or is not) performed, promoting the same intent in both frame types (Levin et al., 1998). Since multiple aspects can be manipulated in message frames, their conceptualization is more complex than other framing types, and this increases variability in message frame designs. Hence, in the following, the defining aspects of a message frame are briefly explained. First, a *frame* is defined by the consequences (gain or loss) and the execution (executed or not executed) of an action or behavior. This reasoning results in a 2 × 2 matrix that defines four possible frame types within message framing: (A) obtain a gain when behavior is executed; (B) avoid a loss when behavior is executed; (C) forego a gain when behavior is not executed; and (D) suffer a loss when behavior is not executed (see Table 1). These four frame types can be differentiated by their value (*frame value*) being either positive (A and B) or negative (C and D). The contrast between the frame types A and D is assumed to be the most extreme and consequently most successful in producing a message framing effect (Levin et al., 1998). This so-called pure cross-complement message framing is used in this research study.

**Table 1:** Frame types of message framing.

	**Execution | Behavior X**	**Non-execution | Behavior not-X**
Positive frame	(A) Obtain a gain when behavior is executed	
	(B) Avoid a loss when behavior is executed	
Negative frame		(C) Forego a gain when behavior is not executed
		(D) Suffer a loss when behavior is not executed

A frame is defined by the consequences (gain or loss) and the execution (executed or not executed) of an action or behavior.

Negations are regularly used to formulate the negative frame value (Levin et al., 1998) by saying “If behavior X is not performed, then a loss will occur,” resulting in a formulation that *indirectly* reflects the supporting intent of the argument. Complementarily, the non-negated message formulation for positive frame values *directly* explicates the consequences that follow from the behavior (A and B). The way information is phrased can have implications for how it is processed, as will be discussed later in this article.

In addition to these aspects of message framing design, previous meta-analyses investigating the persuasiveness of message framing on sustainable consumer behavior and health-related issues suggest that there might also be some variations within the *object*, i.e., the action or behavior being framed (Ainiwaer et al., 2021; Florence et al., 2022). The object to be framed might not always be perceived positively and, especially in health communication and environmental issues, negatively valued objects (such as screening for cervical or colorectal cancer or cumbersome procedures associated with sustainable behavior) might be the object of message framing communication (Gallagher and Updegraff, 2012; Ainiwaer et al., 2021; Florence et al., 2022). For these objects, the meta-analytic results are inconclusive, showing either no effects of the frames, a higher persuasiveness of positive frames (Gallagher and Updegraff, 2012; Florence et al., 2022), or sometimes a higher persuasiveness for negative frames (Ainiwaer et al., 2021; Florence et al., 2022). As a possible property that could explain these effects, this study tests the value of the object as the general public preconception toward the object (*object value*), which can be either positive or negative.

Indications of how different object values might affect the persuasive impact on consumer behavior are provided by studies on emotions in the context of message framing (Nabi et al., 2020). It is suggested that emotions can form a frame in themselves (emotions-as-frame) through which incoming stimuli are interpreted. A comprehensive meta-analysis reviewed the literature on this topic over the past 30 years, finding a bi-directional influence of emotions on frames and vice versa. More precisely, the frame value elicits congruent emotional responses, meaning that positive (gain) frames elicit positive emotions and negative (loss) frames elicit negative emotions. Whereas, the experience of emotions influences the persuasiveness of corresponding frames, with positive (gain) frames being reinforced by positive emotions and negative (loss) frames by negative ones. In the case of object value, it could be argued that the object value also acts as a frame eliciting an emotional response that subsequently influences information processing. More precisely, a positive object value would be assumed to elicit a general *approach* orientation and a negative object value a general *avoidance* orientation as the emotional frame for further processing.

After defining message framing with the elements *frame value* and *object value, two* different types of frame–object value combinations can be derived that are either consistent (frame and object have the same value) or inconsistent (frame and object have different values). Thereby, a general *approach* or *avoidance* orientation is assumed due to the object value, and the frame value is determined as either a *direct* or *indirect* formulation. To clarify the definitions of the message framing used in this study, the frame types are explicated in the following with an example:

- Direct approach value consistency

° A message that describes the gain that is achieved (*frame value: positive*) if a positively valued object (*object value: positive*) is executed.
° Example: In organic livestock farming, animals have free range, which allows them to perceive their natural environment.

- Indirect approach value inconsistency

° A message that describes the loss that is suffered (*frame value: negative*) if a positively valued object (*object value: positive*) is not executed.
° Example: If in organic livestock farming the animals did not have free range, then they would be limited in their habitat.

- Direct avoidance value inconsistency

° A message that describes the gain that is achieved (*frame value: positive*) if a negatively valued object (*object value: negative*) is executed.
° Example: In conventional livestock farming, the feeding and environment of the animals are monitored, which allows for a high level of food safety.

- Indirect avoidance value consistency

° A message that describes the loss that is suffered (*frame value: negative*) if a negatively valued object (*object value: negative*) is not executed.
° Example: If in conventional livestock farming the feeding and environment of the animals were not monitored, then this would lead to higher health risks.

Using these four frame types, it is investigated in this study how perceived emotional persuasion originates as an emotional response that is experienced when a negative object value is accompanied by a negative compared to a positive frame value; and similarly, a positive (compared to a negative) frame value is used for positive object value. In this regard, a neuroscientific-based model is suggested in this study aiming to describe the underlying processing mechanisms.

### 2.2. A neuroscientific dual-process theory to explain processing during message framing

#### 2.2.1. The reflective-impulsive model

The reflective-impulsive model (Strack and Deutsch, 2004, 2006) builds upon other integrative-generalized dual-process models and incorporates neuroscientific evidence into its theoretical considerations, like the neuroscience-based reflexion-reflection model (Smith and DeCoster, 2000; Lieberman et al., 2002; Strack and Deutsch, 2004).

The basic assumption of this integrative dual-process theory is that there are two separate and interacting processing types operating in parallel—impulsive and reflective (Strack and Deutsch, 2004, 2006). The impulsive processing type processes information through associations via distributed activation and establishes connections gradually based on the principles of contiguity and similarity (Strack and Deutsch, 2004, 2006). The impulsive processing type is guided by an approach and avoidance orientation, which can be triggered either by processing positive or negative *affective information*, or by experiencing a corresponding affect, or by perceiving or performing approach and avoidance behaviors (Gray, 1982; Lang, 1995). The reflective processing type is assumed to guide a decision-making process that is based on deliberation and integration processes, requiring *cognitive capacities* (Strack and Deutsch, 2004, 2006). This processing type generates semantic connections between elements by assigning values via propositional categorization and syllogistic inference. During this process, the reflective processing type can hold a limited amount of information for some time in the working memory to assign semantic meaning. Both processing types work in parallel and exchange information; for example, the reflective processing type activates non-associated elements within the impulsive processing type. As a result of the processing, it is assumed that the impulsive processing type generates an experiential “gut feeling” awareness and the reflective processing generates a noetic “sense of knowing” awareness (Strack and Deutsch, 2004).

A relevant feature in this study is that the processing principle of the reflective processing type also allows the understanding of negations, reversing value propositions, which the impulsive processing type cannot process such that it can only extract the information as a non-negated concept. To illustrate this, an example: for the indirect avoidance value-consistent message “If in conventional livestock farming the feeding and environment of the animals were *not* strictly monitored, *then* this would lead to higher health *risks*,” the reflective processing type is assumed to process the negative consequences as supportive for the object, since they would occur if the conventional farming act were not performed. However, the impulsive processing type is not supposed to be able to process the clause that the negative consequence (higher health risks) only occurs for not executing the act. It is expected that only the negative consequence is processed, and that is associated with the negative object value (conventional livestock farming). That the processing of negation is more complex and requires more cognitive resources is supported by multiple studies suggesting that negation slows down cognition and is more error prone, which would indicate, according to the model, that the reflective processing type is operating (Wason and Jones, 1963; Wegner et al., 1985; Strack and Deutsch, 2004).

#### 2.2.2. Network perspective of emotion–cognition interaction

In general, this model seems to be a framework that has so far been able to explain many phenomena and to integrate other theories and hypotheses that take into account neuroscientific findings (Higgins, 1997; Smith and DeCoster, 2000; Lieberman, 2007). Moreover, neural investigation seems essential in the context of message framing, since emotions appear to be influenced in both directions by frames and objects, making it almost impossible to distinguish cause and effect when evaluating from self-reports. A better understanding of unconscious and emotionally influenced processes would be necessary and could be achieved with the help of neuroscientific methods (Karmarkar and Yoon, 2016). Nonetheless, the applied model should be considered as an organizing framework that allows us to structure the types of processing that are thought to occur in decision-making during message framing. However, these processing types cannot be defined *per se* in terms of one neural structure, but it should be possible to identify neural correlates that are involved in the interaction of these two processing types. If the proposed processing types are interactively operating, then differential effects at the neural level should be identifiable.

With this assumption, this research study fundamentally adopts the network perspective of the brain (Pessoa, 2012, 2017). The assumption is that a brain region cannot perform a function on its own, but at best integrates signals that are part of a processing network that makes up that function. Rather, brain areas seem to be more or less involved in emotional or cognitive processing. It is assumed that there is a distributed network that neurally represents the interaction between cognitive and emotional processes (Pessoa, 2012, 2017). It consists of a number of different regions involved in the integration of emotion and cognition signals distributed across all brain regions, including both prefrontal and subcortical structures (Pessoa, 2010). These structures include the hypothalamus, basal forebrain, and amygdala as subcortically located brain areas, as well as areas of the prefrontal cortex, such as the cingulate and orbitofrontal cortex, and the insula. All these areas are strongly anatomically and functionally interconnected with other subcortical and cortical brain structures (Pessoa, 2010, 2017). This supports the reasoning that these structures may have the potential to carry out the interactions of emotion and cognition as functionally integrated systems, reflecting the processes suggested in the aforementioned reflective-impulsive model. In the following sections, the functions to which the aforementioned brain areas contribute and the processes associated with them are only briefly described according to the original research, as a more detailed description has already been given elsewhere (Pessoa, 2010).

As already mentioned, the assumed emotion-cognition integrative brain areas can be roughly differentiated into subcortical and cortical structures. Starting with the latter, generally, brain areas related to the prefrontal cortex are mainly associated with cognitive functions, including attention, working memory, decision-making, and other higher-order cognitive processes such as executive function, top-down reasoning, and inhibitory control (Carlén, 2017). Moreover, the subregions cingulate and orbitofrontal cortex seem to be largely connected to subcortical structures (Carmichael and Price, 1996; Vogt and Derbyshire, 2009; Vogt and Vogt, 2009; Klein et al., 2010). They are assumed to function as a hub that links signals with brain stem systems, integrating extensive sensory information into the evaluation process (Lim et al., 2013; Pelletier and Fellows, 2019). This could allow for value inference or comparison processes, being also described as one part of a valuation system to guide decision-making (Bartra et al., 2013).

The anterior insula, however, has a somewhat different, more internal state monitoring function compared with the prefrontal structures (Craig, 2002, 2009). It seems to be critically involved in the processing of bodily signals, providing “afferent representations of ‘feelings' from the body” (Pessoa, 2010, p. 437). With this notion, there is apparent parallelism with the impulsive processing type, which assumes that a kind of “gut feeling” arises during processing by the impulsive processing type.

In contrast to the prefrontal cortex, the subcortical areas, hypothalamus, basal forebrain, and amygdala, constitute evolutionarily older areas (Rolls, 2015; Carlén, 2017). Based on anatomical, neurophysiological, functional neuroimaging, and neuropsychological evidence, an association of these structures is traditionally made with emotions (Pessoa, 2010), all structures being part of the limbic system (Rolls, 2015). However, they are also connected with a multitude of other structures either in the prefrontal cortex or in the brain stem (Young et al., 1994; Agosta et al., 2021). They are assumed to play a critical part in multiple cognitive processes, whereby these areas are associated specifically with the processing of emotional stimuli, the flow of information from the sensory cortex, and conveying emotional information (Pessoa, 2010).

These regions must therefore be involved in some way in the processing of message framing, since here, too, integration of emotional and cognitive processes is assumed. To give an indication of how this integration might take place conceptually, the reflective-impulsive model will be used as a theoretical framework. This allows us on the one hand to explain the behavioral frame-object consistency effect and on the other hand to hypothesize which regions should be observable at least as part of the process.

## 3. Hypothesis derivation

In the following, the processing of message framing is explained against the background of the reflective-impulsive model. First, it is explained how information in message framing are processed in the impulsive and reflective processing types as assumed by the model. To derive the neural hypotheses, some assumptions are made about brain areas that should be activated if their integrative role in the interaction between emotion and cognition is correctly assumed by the network perspective.

### 3.1. The reflective-impulsive interpretation of message framing

The specification of message framing leads to the differentiation of four frame types as explicated with examples in Section 2.1. In light of the behavioral findings on message framing, some simple assumptions can be made about the effects of frame-object consistency. Building on the emotion-as-frame argument, it is argued that the object value induces a corresponding emotional response. If the incoming frame value is consistent with the object value (*value consistency*), the effect of message framing is expected to increase compared with inconsistent frame values (Nabi et al., 2020). Although expectations can be formulated based on previous review articles and meta-analyses on message framing (Gallagher and Updegraff, 2012; Nabi et al., 2020; Xu and Huang, 2020; Ainiwaer et al., 2021; Florence et al., 2022) and the effects on (in-)consistent frame-object values, the underlying processing mechanisms giving rise to these effects is still largely unknown.

Accordingly, in the following, the four different frame types used in this study will be explained based on the processing within the two processing types: impulsive and reflective. According to the model, only the reflective processing type can process negations and extract the propositional meaning from the message, whereas the impulsive processing type is orientated toward approach and avoidance via the motivational orientation elicited by the framed object (Strack and Deutsch, 2004).

We start with the interpretation of the message types for the reflective processing type. In all mentioned messages (direct approach value-consistent; indirect approach value-inconsistent; indirect avoidance value-consistent; direct avoidance value-inconsistent), the reflective processing type can extract the meaning of the message as supportive for the object. In both frame values, the messages provide arguments that support the object. More precisely, in the positive frame benefits are indicated that are achieved when the object is executed. Similarly, in the negative frame, losses or harms are mentioned that are suffered when the object is not executed, thus arguing for the implementation of the object. Since the reflective processing type can decode negations, the negative frame can also be interpreted as supportive information in favor of the object. The message in all frame types creates a sense of knowing that the object should be supported based on the information.

More interesting is the hypothesized processing within the impulsive processing type. First, for the direct approach value-consistent messages, the impulsive processing type is assumed to have an initial approach orientation due to the positive object value. This approach orientation is then confirmed by a positive frame value, resulting in a positive gut feeling (*experiential awareness*). Second, for the indirect approach value-inconsistent messages, an initial approach orientation is elicited in the impulsive processing type due to the positive object value. The subsequent indirect formulation of the negative frame value, naming negative consequences that would occur if the behavior were not executed, conflicts with the initial approach orientation, since the negation is not processed and the message not interpreted as supportive for the object. This results in a negative, conflicting gut feeling associated with this message framing. Third, for direct avoidance value-inconsistent messages, the initial orientation would be avoidance due to the negative object value; however, this time, it is paired with a positive frame value, communicating positive consequences of the object. Similarly, this combination is assumed to create a conflicting experiential awareness and negative gut feeling for this message framing in the impulsive processing type. Fourth and foremost, for the indirect avoidance value-consistent messages, a positive gut feeling is elicited. Again, the initial orientation is avoidance, since the object is perceived as negative. However, this time, the negative frame value confirms the avoidance orientation, resulting in a positive experiential awareness of this message framing.

The interpretation of the four message frames based on the assumptions of the reflective-impulsive model are summarized in Table 2. This interpretation leads to behavioral hypotheses, where the reflective-impulsive model provides an explanation for why these effects occur. Owing to the positive awareness evaluation in both processing types for the consistent frame-object messages, consistent messages should result in a more positive evaluation compared with the inconsistent frame-object combinations. Therefore, the following hypothesis can be assumed:

**Table 2:** Summary of message frame interpretation based on the reflective-impulsive model.

**Object value**	**Positive**				**Negative**			
**Frame value**	**Positive**		**Negative**		**Positive**		**Negative**	
**Formulation**	**Direct**_non − negated_		**Indirect**_negated_		**Direct**_non − negated_		**Indirect**_negated_	
**Processing type**	**Reflective**	**Impulsive**	**Reflective**	**Impulsive**	**Reflective**	**Impulsive**	**Reflective**	**Impulsive**
Message interpretation	Message supports object	Message confirms approach orientation	Message supports object	Message conflicts approach orientation	Message supports object	Message conflicts avoidance orientation	Message supports object	Message confirms avoidance orientation
	✓	✓	✓	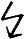	✓	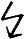	✓	✓
Awareness evaluation	Positive sense of knowing	Positive gut feeling	Positive sense of knowing	Negative gut feeling	Positive sense of knowing	Negative gut feeling	Positive sense of knowing	Positive gut feeling
Message framing	Direct approach value consistency		Indirect approach value inconsistency		Direct avoidance value inconsistency		Indirect avoidance value consistency	

The messages are characterized by the object and frame value as well as the formulation as negated or non-negated statement. The interpretation of the message can either be supportive and confirming or conflicting, creating a specific awareness evaluation.

**H1a**_**behavioral**_: For positively valued objects, positive frames are evaluated more positively than negative frames.

**H1b**_**behavioral**_: For negatively valued objects, negative frames are evaluated more positively than positive frames.

### 3.2. The neuroscientific dual-process interpretation of message framing

Following the reflective-impulsive model, which assumes parallel and interactive processing of the reflective and impulsive processing types, it can be assumed that at least some of the aforementioned integrative emotion-cognition brain areas are active. Based on the reflective-impulsive model, some features can be defined for the interaction that can subsequently be associated with brain areas. As an underlying premise in this research, it is assumed that the integrative interaction between the two processing types is only necessitated if the result of their processing is unambiguous. That is, when each processing type processes the message as conclusively supporting or confirming the initial orientation to the object. Only in cases where both processing types process the message unambiguously, their processing signals need to be integrated. Consequently, inconsistent messages between frame and object should not lead to such integrative interaction. However, their counterparts within the same object value are thought to lead to these integrative processes, which should, at least to some degree, be reflected in the underlying neural brain areas associated with the processes.

For positively valued objects, the theoretical interpretation suggests that a positive frame value leads both processing types (impulsive and reflective) to a positive evaluation of the message compared with a negative frame value. More precisely, the direct approach value-consistent message is evaluated by the reflective processing type as supportive and, likewise, the impulsive processing type interprets this message as confirming the approach orientation. Consequently, both processing types assimilate the message as supportive for the object, resulting in a coinciding processing of the message within both processing types. Hence, areas that incorporate both processing evaluations as an integrative, subjective evaluation should elicit more neural activation compared with the indirect approach value-inconsistent messages, where only the reflective processing type has a supportive evaluation of the message. Brain areas that are associated with the integration of information from prefrontal and interconnected subcortical regions and with evaluation processes are the orbitofrontal and anterior cingulate cortex (Deppe et al., 2005, 2007). It is therefore hypothesized that:

**H1a**_**neural**_: For positively valued objects, an increased neural activation in prefrontal areas, more precisely the orbitofrontal and cingulate cortex, is assumed for direct approach value-consistent messages compared with indirect approach value-inconsistent messages.

Complementarily, for negatively valued objects, indirect avoidance value-consistent messages are expected to be evaluated as supportive by both processing types. Thereby, the reflective processing type processes the message as supportive as, again, the negative consequence is negated in the message, hence only occurring when the action is not performed. However, the impulsive processing type processes the message as confirmative to the avoidance orientation, resulting in a positive experiential feeling that the message matches the orientation but as a counterargument to the object. Hence, both processing types assimilate the message as supportive or confirming, but in opposing directions with regard to the support of the object. Specifically, when comparing the indirect avoidance value-consistent message with the direct avoidance value-inconsistent message, the increased confirmed motivational avoidance orientation with the impulsive processing type seems to be the decisive difference for the indirect avoidance value-consistent message. Consequently, areas associated with the functions of conveying emotional information and integrating “afferent representations of ‘feelings' from the body” (Pessoa, 2010, p. 437) are assumed to be increasingly activated.

**H1b**_**neural**_: For negatively valued objects, an increased neural activation in the insula and amygdala is assumed for indirect avoidance value-consistent messages compared with direct avoidance value-inconsistent messages.

## 4. Method

To study the effect of message framing for different objects, a topic needed to be selected that would enable the creation of positive and negative object values while still being in the same thematic sector. Considering the need to foster communication for behavioral change as a motivation for this research, this study focused on the example of livestock farming as a sustainability issue (UN, 2015; Mehrabi et al., 2020; Schneider and Tarawali, 2021). The differentiation of livestock farming approaches allowed us to apply the message framing on the same topic but for two different object values. Thereby, aspects of farming production in two types of livestock farming (conventional and organic) were used as objects stimulating either positive or negative object values. Thereby, organic livestock farming approaches should function as a presumably positive object value and conventional livestock farming as a presumably negative object value (Christoph-Schulz et al., 2015).

Livestock farming is a complex and widely differentiated sector, with multiple approaches to how farm animals should be reared, and is the matter of ongoing discussion (e.g., European Commission, 2005, 2016; Krystallis et al., 2009). Consequently, the perspectives of how to communicate about livestock farming are sometimes strongly divergent (Busch and Spiller, 2018; Rovers et al., 2019; Schütz et al., 2022). It should be noted that appropriate communication about livestock is not the aim of this study. This research aims to understand the processing of message framing effects on a more abstract, fundamental, and general level. To create an effective study design for this aim, the different livestock farming approaches and farming aspects were strongly abridged. To capture a general impression of the chosen topic (livestock farming) and differentiate the effects on value consistency, messages were formulated for two livestock farming approaches that were assumed to serve consumers' prejudices and were perceived to be in opposition to each other (Ismael and Ploeger, 2020).

### 4.1. Procedure

To test the hypotheses, an fMRI study was conducted in Germany. Ethical approval for the conducted study was given by an ethical committee. After being welcomed, the participants were informed about the study and its procedure before they signed the written informed consent. Within the MRI scanner, participants were equipped with ear protection, MRI-compatible goggles, and the input device that was used to complete the task within the scanner. After adjustments of the scanner, two test trials of the experimental task were completed, during which the participants could practice the handling of the input device and get used to the task design. The experimental task took about 45 minutes. Including the structural scan, participants stayed overall 1 h in the MRI scanner. At the end of the experiment and outside the MRI scanner, participants completed a questionnaire that included control variables, such as dietary habits, consumption of animal products, and attitudes toward animals in general, and provided demographic information. Subsequently, participation was completed and participants received monetary compensation.

### 4.2. Participants

In total, 32 participants were recruited via an institutional participant pool for this study (*N* = 32), ensuring no convenience sampling of students only. Owing to extensive movement (translation of ± 3 mm) during the fMRI scanning procedure, three participants had to be removed from the data analysis, resulting in a final sample size of *n* = 29. The participants were aged between 20 and 56 years (*M*_age_ = 41.45, *SD*_age_ = 10.83) and consisted of 14 women and 15 men. Participants were right-handed and within the normal weight range (BMI 19–26). Exclusion criteria were applied, including pregnancy, diabetes, drug dependence, smoking, cardiovascular diseases, and psychological or neurological diseases, as well as fMRI-related exclusion criteria (e.g., claustrophobia, metal implants, tattoos, or permanent make-up). All the criteria were assessed via a pre-screening questionnaire and fMRI-related criteria were inquired about once again before the scanning session.

In addition to these method-related criteria, other control variables were recorded regarding the topic under investigation (livestock farming), to better describe the sample. In this regard, no vegetarians or vegans were recruited to prevent confounding effects that might occur due to their intensified moral reservations concerning the selected topic (Bennett et al., 2002; de Jonge et al., 2015). Most of the included participants stated that they consume meat several times per week (86.2%). When asked if they pay attention to organic production when buying meat, 79.3% said they are moderately to definitely attentive, although the question and responses raise the question of a social desirability bias (Norwood and Lusk, 2011). Furthermore, participants seemed to place moderate importance on meat purchases (*M*_CIP_ = 3.6, *SD*_CIP_ = 1.09, measured with a modified importance subscale of the consumer involvement profile on a 5-point Likert scale; Laurent and Kapferer, 1985) and had a moderate positive attitude toward animals in general (*M*_AAS_ = 3.81, *SD*_AAS_ = 0.59, measured with the animal attitude scale on a 5-point Likert scale; Herzog et al., 1991).

### 4.3. Materials

#### 4.3.1. Stimuli design

Messages of two different object values (positive and negative) were formulated in two frame values (positive and negative). For each of the different object values, two frames were created, either expressing a positive or negative frame value. First, aspects of livestock farming production were identified that should be supported by the arguments provided in the message framing. Therefore, basic messages were formulated that included only the description of the livestock farming aspects without any further explanatory (frame) information, e.g., “In conventional livestock farming, the feeding and environment of the animals is monitored.” Then, the frames were created, adding explanatory information. For positive frame values, the gains received from the object when it is executed were explained, e.g., “In conventional livestock farming, the feeding and environment of the animals is monitored, which allows for a high level of food safety.” In contrast, for negative frame values, potential harms that could be suffered if the object is not performed were explained, e.g., “If in conventional livestock farming the feeding and environment of the animals were not monitored, then this would lead to higher health risks.” Thereby, the negative frame values were formulated as a negated, indirect statement. Furthermore, pictures illustrating the livestock farming aspect were selected. In an online pretest (*N* = 84, 41 women, 43 men, *M*_age_ = 40.79 years, *SD*_age_ = 14.72), all messages were evaluated on credibility and valence on a 10-point semantic differential scale. Furthermore, out of a collection of three pictures, participants rated the most suitable one for each livestock farming aspect.

Results of pretest on the valence evaluation confirmed that the two object values (conventional = negative and organic = positive) were perceived as intended. Aspects of organic livestock farming were evaluated as significantly more positive than conventional livestock farming [*t*_(83)_ = −12.468, *p* < 0.001; *M*_organic_ = 7.54, *SD*_organic_ = 1.42, *M*_conventional_ = 4.49, *SD*_conventional_ = 1.96]. Furthermore, the messages with the highest credibility and most selected pictures were chosen for the experiment (four per animal type: cow, pig, and chicken). This resulted in 12 messages per object value for the experimental task, resulting in a total of 24 messages used in the experimental task.

#### 4.3.2. Experimental task

**Figure 1:**
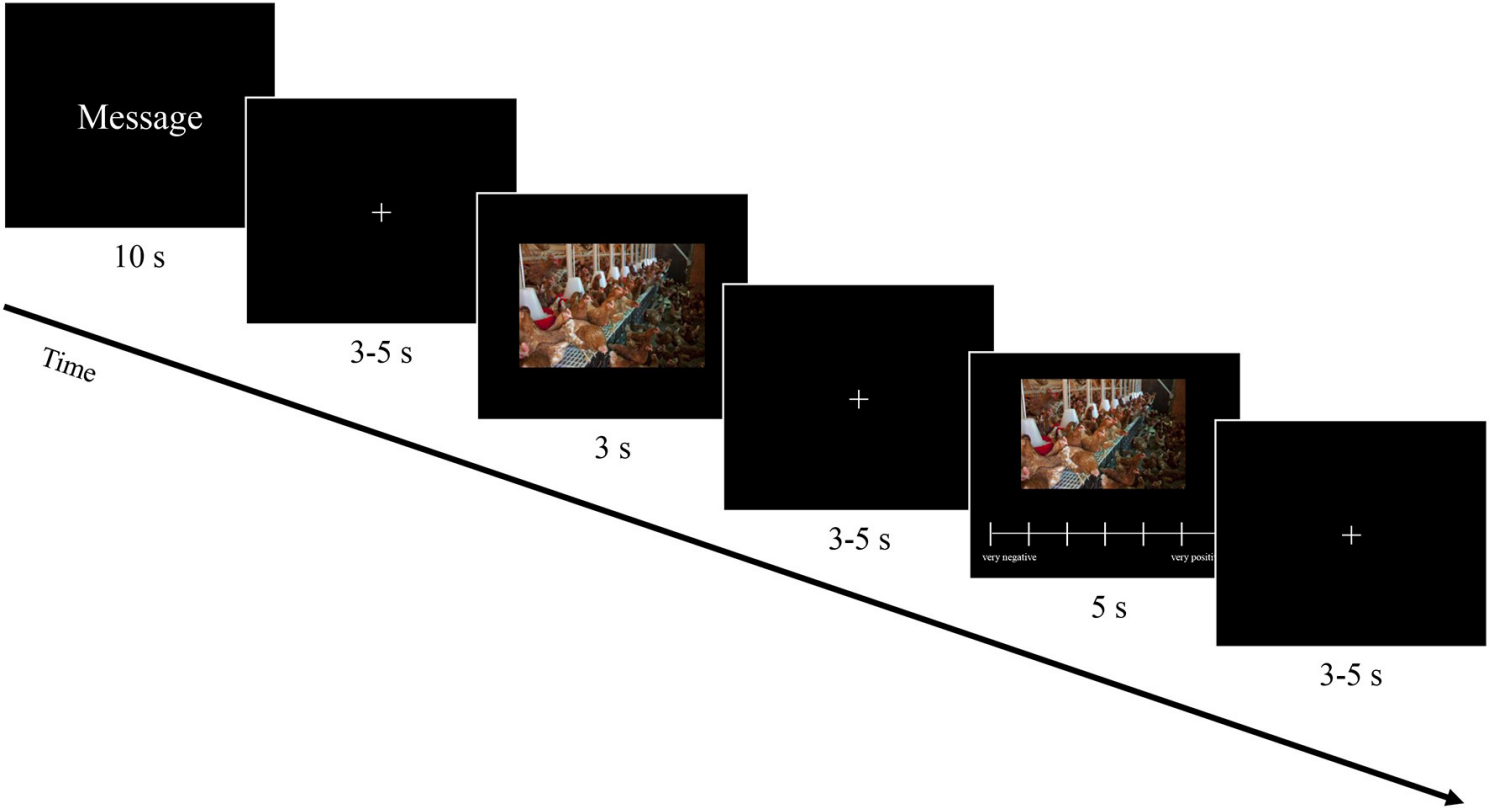
Trial sequence of the experimental task. A trial consisted of three parts, each separated by a randomized intertrial interval (3–5 s). First the message was shown (10 s), which was either basic “In conventional chicken farming, the chicken are kept in sheds,” positive frame value “In conventional chicken farming, the chicken are kept in sheds, which offers a high level of safety,” or negative frame value “If the chickens in conventional chicken farming were not kept in sheds, there would be a risk of contamination.” Thereafter, a corresponding picture was shown (3 s) and subsequently the picture was evaluated (5 s).

The experimental task in the fMRI consisted of three blocks. In the first and the last blocks, the 24 basic messages were displayed in a randomized order, including only the description of the livestock farming aspects. In the first block, the intended prejudiced, socially desirable approach/avoidance orientation was to be stimulated to increase the perceived difference between the object values. In addition, these blocks should specifically enable identification of a possible change beyond the framing in the evaluation of the livestock farming aspect. The second block included the message framing with 48 trials (24 per frame value), which were also displayed in randomized order. Every block started with a short task description, saying that the participant should attentively read the message, look at the picture, and enter the evaluation via the input device. A trial started with the presentation of the message shown for 10 seconds (s). To keep the number of words and, therefore, the perceptual load similar, every message consisted of two lines of text for the basic messages and three lines of text for the message framing. Messages were displayed in white letters centered on a black screen. After an interstimulus interval (ISI) displaying a fixation cross in the center of the screen with a random duration (3–5 s; jitter), the associated picture was shown (3 s) followed by another ISI jitter (3–5 s). At the end of each trial, participants had to rate the picture on a 7-point Likert scale, ranging from very positive to very negative, while the picture was still displayed. Pictures were used as rating stimuli as the aim was to compare the effect of message framing on the processing and evaluation of equal stimuli. To ensure that the evaluated content was the same and only the message framing was manipulated between the conditions, the evaluated stimuli were kept fixed using the associated pictures of the livestock farming aspect. This allowed for a methodologically clean interpretation of the behavioral effects. The next trial started after participants entered and confirmed their rating followed by an intertrial interval in the form of a jitter (3–5 s). If no answer was given, the trial continued automatically after 5 s. These trials were later entered as missing in the data analysis. In total, the tasks included 96 trials and took ~45 min. A schematic trial sequence is shown in Figure 1.

### 4.4. Data analysis

#### 4.4.1. Behavioral data analysis

To analyze the behavioral effects of the message framing during the fMRI experiment, a repeated measurement analysis of variance (RM-ANOVA) was executed. The ratings on the 7-point Likert scale during the evaluation period of the experimental task were entered as the dependent variable into the analysis. As independent within-subject variables, the block/frame value (basic first block, basic last block, positive and negative frame value) and object value (positive and negative object value) were included, resulting in eight within-subject factor conditions from the 4 × 2 experimental design. Prerequisites of RM-ANOVA were confirmed by statistical analyses using the Shapiro–Wilk test for normality and Mauchly's test of sphericity. The Shapiro–Wilk test indicated a non-significant result for three out of the eight within-subject conditions. Hence, five conditions were assumed to be non-normally distributed on the dependent variable. Even though normality assumption violations were less problematic compared to homogeneity, the skewness in these conditions was further inspected (Blanca Mena et al., 2017). The skewness statistics (standardized skewness coefficient and associated standard error) indicated that by the rule of thumb (± 2 std. error, coefficient within this range; Leech and Onwuegbuzie, 2002), only the within-subject conditions of gain frames in the negatively valued object could still be considered as problematic. Visual inspection of the distribution suggested one outlier. Repeating the analysis excluding the outlier resulted in no normality violations according to the rule of thumb. However, as the analysis without the outlier yielded comparable results in effect size and *p*-values, the analysis of all *n* = 29 cases is reported to increase power and ensure comparability with the neural results.

Mauchly's test of sphericity revealed an inequality of variance between the within-subject conditions for the overall RM-ANOVA (*W* = 0.059, χ^2^ = 72.45, *p* < 0.001), as well as the follow-up analysis on the RM-ANOVA for the two object values separately (positive object value: *W* = 0.142, χ^2^ = 52.184, *p* < 0.001; negative object value: *W* = 0.585, χ^2^ = 14.35, *p* = 0.014). Consequently, Greenhouse–Geisser corrected results are presented.

#### 4.4.2. fMRI acquisition

Functional brain images were obtained with a 1.5 Tesla Siemens Avanto Scanner (Erlangen, Germany). An echoplanar imaging sequence was used with a repetition time of 2.5 s, an echo time of 45 ms, and a flip angle of 90°. A brain volume contained 31 slices assessed in a regular-up pattern with axial orientation. Each slice had a thickness of 3 mm and an interslice gap of 0.3 mm. Experimental tasks were programmed with a scanner-institute-internal task designer tool, based on the programming language Python. Participants wore MRI-compatible goggles to see the tasks within the scanner (NordicNeuroLab VisualSystem, Bergen, Norway). Responses could be entered via two input devices with the right and left hands of the participants. Thereby, the participants could shift the response on the scale with their two index fingers (right = more positive; left = more negative) and confirm their answer by pressing the button with their right thumb.

The fMRI data were pre-processed utilizing the processing function of SPM12 software package (Wellcome Department of Imaging Neuroscience, London, UK) implemented in MATLAB (The MathWorks, Inc.; R2016a). Images were slice time and motion corrected. A generalized field map, acquired before the experimental scanning, was used for unwarping the functional images. The participants' brains were normalized to the Montreal Neurological Institute (MNI) standard brain. Images were resliced to 3-mm-isotropic voxel size and smoothed with an 8-mm full-width-half-maximum Gaussian kernel. Additionally, a 127-s high-pass filter was applied for temporal filtering.

#### 4.4.3. fMRI data analysis

fMRI data analysis was done with SPM12 in MATLAB. For every participant, a general linear model (GLM) was set up to model neural activity during the experimental task. Message period, picture period, and evaluation period were modeled separately for each block/frame value (basic first block, basic last block, and positive and negative frame value) and object value (positive and negative object value), adding up to 24 event-related regressors. Evaluation events were aligned to the time of response. Trials of no response were modeled as misses separately for each period, resulting in three additional regressors. Using the parameters from the motion correction of the pre-processing, six movement regressors were included in the GLM together with a constant term. Each time course was convolved by a hemodynamic response function. Two contrasts of interest were calculated for every participant and later entered in the group analysis. Thereby, consistent frame-object messages were contrasted with their inconsistent frame-object messages per object value. More precisely, message periods of the positive vs. negative frame values were contrasted for positive object values to evaluate H1a _neural_. To evaluate H1b _neural_, the message periods of negative vs. positive frame values were contrasted for negative object values. Inverse contrasts for each object value were analyzed as well. Using the individual contrast maps, *t*-maps were calculated in the group analysis. Regions of activation were identified using a statistical threshold of *p* < 0.05 with whole-brain false discovery rate (FDR) correction and a minimum cluster size of 20 voxels. Activation maps, statistics, and anatomical labeling were done by the xjView toolbox (http://www.alivelearn.net/xjview).

## 5. Results

### 5.1. Behavioral results

Regarding the manipulation check, positive object values were indeed evaluated more positively than negative object values [*M*_organic_ = 5.86, *SD* = 0.4732; *M*_conventional_ = 2.617, *SD* = 0.6399; *F*_(1, 28)_ = 392.945, *p* < 0.001, $\eta_{\text{p}}^{2}$ = 0.933], as identified by a significant main effect of object value. Thereby, the perceived difference between the object values, as seen in the mean differences, was intensified compared to the pretest results. This indicates that the experimental task enhanced the intended biased, socially desirable approach/avoidance orientation in the desired manner. More interestingly, a significant interaction effect between block/frame value and object value could be identified [*F*_(1.389, 38.879)_= 38.667, *p* < 0.001, $\eta_{\text{p}}^{2}=$ 0.58].

**Figure 2:**
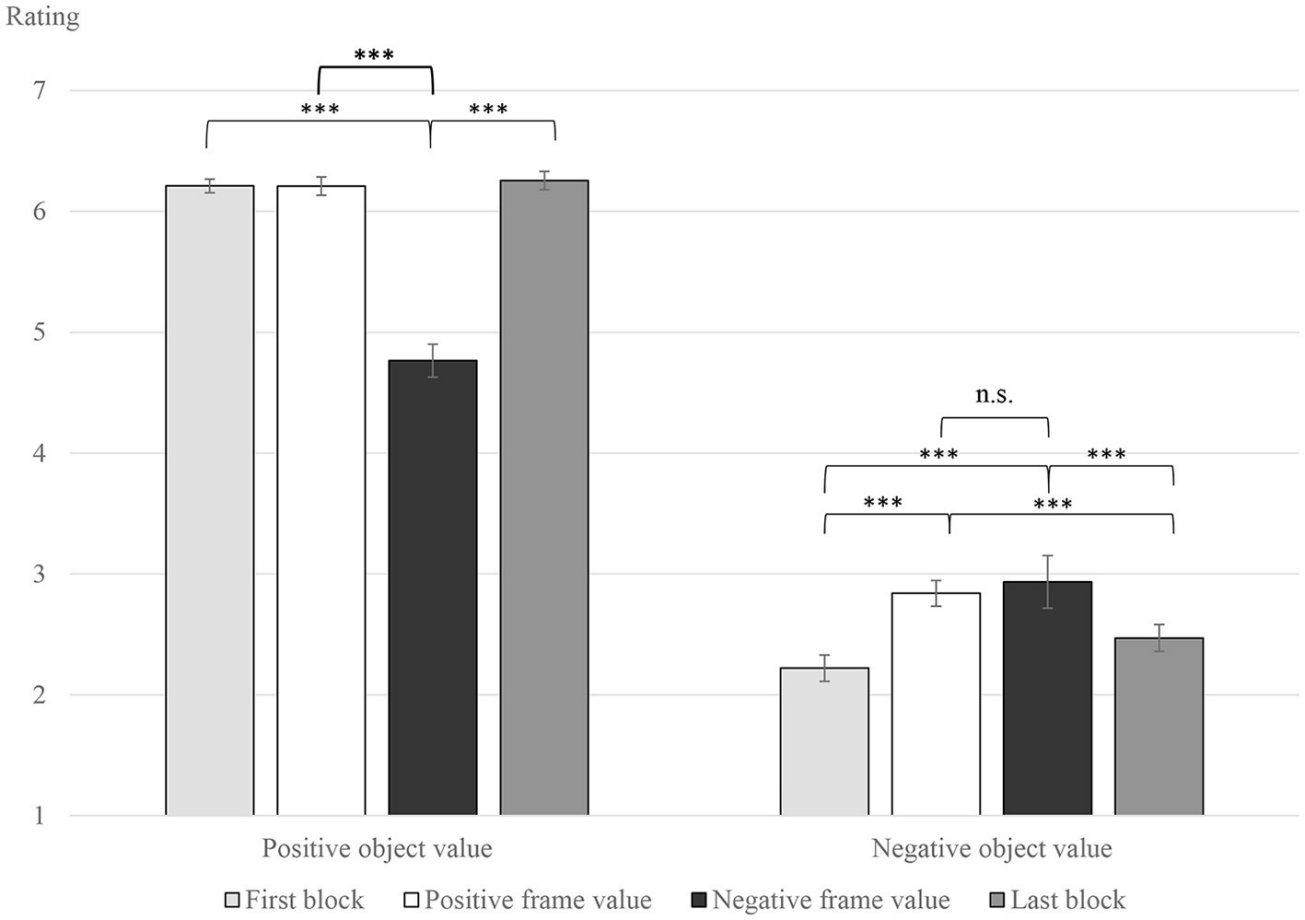
Mean values of the evaluation rating per block/frame value and object value. For positive object values, negative frame values significantly decreased the evaluation. Both frame values significantly increase the evaluation of negative object values compared to basic messages. Error bars indicate standard error of the mean. ***p < 0.001; n.s., non-significant.

As *post-hoc* analyses, two separate RM-ANOVAs per object value were executed. The mean values of the ratings and significant differences can be seen in Figure 2. The two analyses confirmed that the rating per block/frame value significantly differed for both positive and negative object values [positive object value: *F*_(1.241, 34.747)_= 30.548, *p* < 0.001, $\eta_{\text{p}}^{2}=$ 0.522; negative object value: *F*_(2.280, 63.847)_ = 20.007, *p* < 0.001, $\eta_{\text{p}}^{2}$ = 0.417].

Analyzing positive object values, ratings after negative frame values (*M*_negative − frame_ = 4.765, *SD* = 1.174) were significantly more negative than ratings for positive frame values [positive frame value: *M*_positive − frame_ = 6.209, *SD* = 0.577; *t*_(28)_ = 5.932, *p* < 0.001], confirming H1a _behavioral_ as well as the basic blocks [basic first block: *M*_basic − first_ = 6.211, *SD* = 0.586; *t*_(28)_ = 5.339, *p* < 0.001; basic last block: *M*_basic − last_ = 6.256, *SD* = 0.6; *t*_(28)_ = −6.148, *p* < 0.001].

Comparing the consumers' ratings for messages on negative object values, both frame values (*M*_positive − frame_ = 2.84; *SD* = 0.781; *M*_negative − frame_ = 2.935, *SD* = 0.841) significantly increased the evaluation compared to the basic messages in the first block [*M*_basic − first_ = 2.222, *SD* = 0.511; compare with positive frame value: *t*_(28)_ = −5.747, *p* < 0.001; compare with negative frame value: *t*_(28)_ = −5.34, *p* < 0.001] and the last block [*M*_basic − last_ = 2.47, *SD* = 0.732; compare with positive frame value: *t*_(28)_ = 4.915, *p* < 0.001; compare with negative frame value: *t*_(28)_ = 3.99, *p* = 0.003]. However, besides a small descriptive difference, there was no significant difference in evaluation between the two frame values for negative object values. Consequently, H1b _behavioral_ had to be rejected, even though a small behavioral tendency could be identified within the descriptive statistics.

### 5.2. Neural results

**Figure 3:**
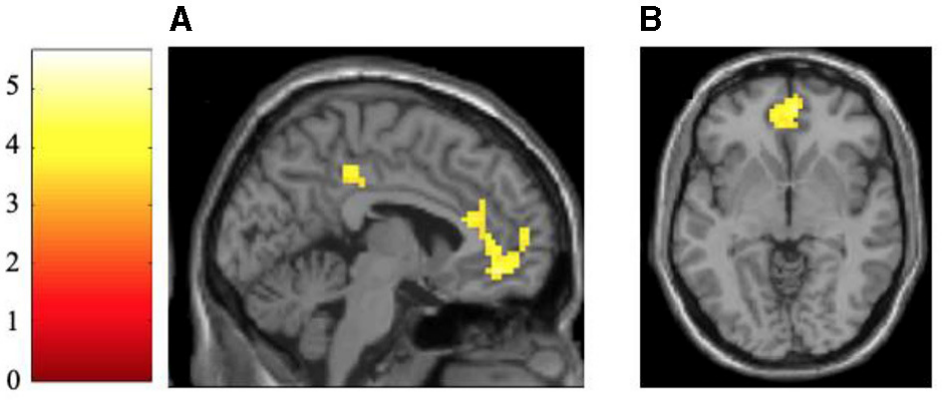
Activation maps of significant activations of the contrast of between direct approach value-consistent messages with indirect approach value-inconsistent messages. The brain images are shown in a sagittal [x = −4 mm; **(A)**] and transversal [z = −4 mm; **(B)**] view, showing the activation in the cingulate medial frontal gyrus as well as the parietal lobe. All activation maps are FDR-corrected at *p* < 0.05 and a cluster size of *n* = 20. The *t-*values are indicated with the color bar.

To test H1a _neural_, the contrast between positive and negative frame values was calculated for positive object values. Results revealed five significant brain activity changes on the voxel level when contrasting direct approach value-consistent messages with indirect approach value-inconsistent messages. Two of these activity changes could be observed in the prefrontal cortex, including the medial frontal gyrus and the anterior cingulate, confirming H1a _neural_. The other three activity changes were mainly located in the parietal or occipital lobe. Details on the localization and activation level can be taken from Table 3 and Figure 3.

**Table 3:** Statistics of significant activations of the contrast between direct approach value-consistent messages with indirect approach value-inconsistent messages.

**Cluster size**	**Area**	***q*_FDR − corr_**	***t*-value**	**x**	**y**	**z**
**204**	**Medial frontal gyrus**	0.026	5.60	3	47	−7
156	Parietal lobe	0.026	5.66	−42	−22	41
23	Occipital lobe	0.038	4.77	−45	−67	−1
**102**	**Cingulate gyrus**	0.041	4.68	3	−31	38
22	Parietal lobe	0.046	4.19	60	−31	44

Reported results are FDR-corrected at *p* < 0.05 and a cluster size of *n* = 20. The localization is indicated with MNI coordinates. The bold values are of interest for the hypotheses.

**Figure 4:**
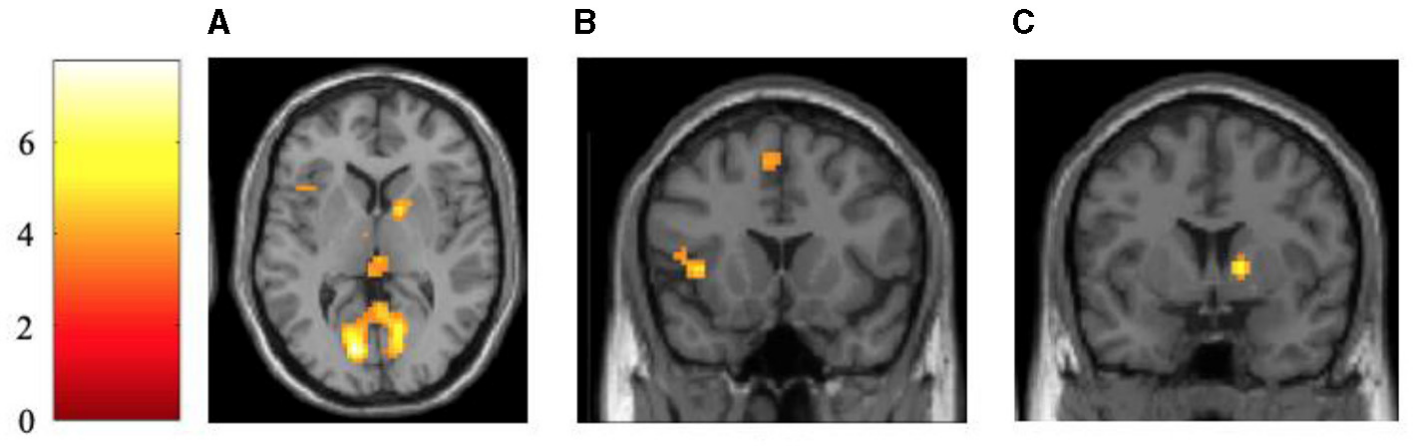
Activation maps of significant activations of the contrast between indirect avoidance value-consistent messages and direct avoidance value-inconsistent messages. The brain images are shown in a transversal [z = 4 mm; **(A)**] and coronal [y = 16 mm; **(B)** and y = 4 mm; **(C)**] view, showing the activation in limbic structures, the insula, as well as occipital lobe. All activation maps are FDR-corrected at *p* < 0.05 and a cluster size of *n* = 20. The *t*-values are indicated with the color bar.

For negative object values, a significant increase in brain activity was hypothesized in the insula and amygdala. To test H1b _neural_, the contrast between indirect avoidance value-consistent messages and direct avoidance value-inconsistent messages was calculated. In total, eight significantly increased neural activations on the voxel level were identified when showing negative frame values compared with positive frame values for the negative object value. Thereby, a significant increase was identified in the insula, partially confirming H1b _neural_. Three out of the eight clusters around the significant peak voxels were located in the subcortical areas, including thalamic midbrain structures. The others were mainly located in the parietal or occipital lobe. Details on the localization and activation level can be taken from Table 4 and Figure 4. Inverse contrasts for each object value are reported in Table 5.

**Table 4:** Statistics of significant activations of the contrast between indirect avoidance value-consistent messages and direct avoidance value-inconsistent messages.

**Cluster size**	**Area**	***q*_FDR − corr_**	***t-*value**	**x**	**y**	**z**
622	Occipital lobe	< 0.001	7.77	−12	−79	5
**27**	**Caudate right/ Putamen**	0.003	5.43	15	5	2
43	Parietal lobe	0.007	5.03	12	−64	44
**32**	**Insula left**	0.008	4.89	−36	17	2
49	Occipital lobe	0.012	4.52	−27	−58	−7
**20**	**Midbrain/ Thalamus**	0.016	4.30	−6	−7	−1
**26**	**Corpus Callosum/ Thalamus**	0.018	4.24	3	−25	5
28	Motor area	0.019	4.21	−3	20	56

Reported results are FDR-corrected at *p* < 0.05 and a cluster size of *n* = 20. The localization is indicated with MNI coordinates. The bold values are of interest for the hypotheses.

**Table 5:** Statistics of significant activations of the complementary contrast.

**Cluster size**	**Area**	***q*_FDR − corr_**	***t*-value**	**x**	**y**	**z**
157	Inferior frontal gyrus	0.002	6.75	−36	2	47
351	Inferior frontal gyrus	0.002	6.42	−45	26	−1
78	Temporal lobe	0.003	5.65	−57	−37	−1
63	Occipital lobe	0.006	5.10	−9	−85	8
52	Motor area	0.016	4.32	−3	14	50

Only significant activations can be identified for loss compared to gain framed message for positive valued objects. No significant activations can be found for gain compared to loss framed message for negative valued objects. Reported results are FDR-corrected at *p* < 0.05 and a cluster size of *n* = 20. The localization is indicated with MNI coordinates.

## 6. Discussion

The aim of this research was to apply an integrative neuroscience-based theoretical approach (i.e., reflective-impulsive model) to explain the effects of message framing on consumer level. The results of the fMRI data analysis indicated different types of neural processing as expected. Behavioral results confirm that for positive object values, positive frame values were evaluated more positively than negative frames (H1a _behavioral_). More specifically, with indirect approach value-inconsistent messages, the evaluation of the livestock farming aspects was significantly reduced compared with the direct approach value-consistent messages and messages without additional framing. This effect confirms the behavioral consequence assumed by prior review articles and meta-analyses on message framing (Gallagher and Updegraff, 2012; Nabi et al., 2020; Xu and Huang, 2020; Ainiwaer et al., 2021; Florence et al., 2022) and suggests the processes assumed within the two processing types. While for positive object values the reflective processing type decodes the negative frame values as supportive, the impulsive processing type perceives a conflict with the initial approach orientation. This conflict results in a negative experiential feeling from this message, reducing the evaluation rating.

However, the hypothesized effects for negative object values could not be supported by the behavioral results, as no significant increase in evaluation rating was identified for negative frame values compared with positive ones (H1b _behavioral_). Still, compared with basic messages before and after the message framing, significant increases for the two frame values could be identified. This indicate that for negative object values, any kind of additional information explaining the livestock farming aspect could help to increase understanding of the object.

More importantly, however, the two object values were assumed to involve different neural brain areas that incorporate affective and cognitive processes in the decision-making process, and thus there could be a potential interaction between processing types, as suggested by the reflective-impulsive model. First, for positive object values, increased neural activation was identified in the anterior cingulate cortex and medial frontal gyrus. These brain areas are assumed to reflect the process of integrating affective and cognitive processes into a valuation process (Bartra et al., 2013). More specifically, the anterior cingulate cortex and more medially located prefrontal brain areas are assumed to function as a hub (Lim et al., 2013; Pelletier and Fellows, 2019), linking cortical prefrontal structures with subcortical areas (Carmichael and Price, 1996; Vogt and Derbyshire, 2009; Vogt and Vogt, 2009; Klein et al., 2010). The medial prefrontal brain areas are often correlated with the function of calculating a subjective value to guide decision-making via value inference or comparison processes (Bartra et al., 2013; Lim et al., 2013; Pelletier and Fellows, 2019).

For negative object values, the neural results indicated that more affective representations from bodily experiential processes seem to be incorporated into the information processing flow when negative frame values are compared with positive frame values. When comparing indirect avoidance value-consistent messages with direct avoidance value-inconsistent messages, increased activation was identified in the insula as well as caudate/thalamic structures, areas associated with affective processing to high-level cognition (Uddin et al., 2017). These activations for the different contrasts provide indications of the potential interaction of different affective and cognitive processing types that might be explicated by the reflective-impulsive model.

In addition to the hypothesized neural effects, significant activity of parietal and occipital brain areas was identified in both contrasts. Occipital brain areas are involved in visual processing (Grill-Spector et al., 1998). Given the increased activity, it is therefore assumed that visual inspection was increased during the processing of value-consistent messages (positive frame value for positive object value and negative frame value for negative object value). Furthermore, activation in parietal areas is associated with memory retrieval and understanding intention (Fogassi et al., 2005; Wagner et al., 2005). One possible interpretation of the increased activation in these areas for value-consistent messages is that evidence is accumulated and retrieved to generate or support the interpretation of the message. This would also support the emotion-as-frame hypothesis (Nabi et al., 2020), which assumes that the experience of an emotion (as approach or avoidance) could guide subsequent information processing. Fundamental neuroscientific research supports this function of evidence accumulation within parietal and occipital lobes, specifically for visual decision scenarios (Mazurek et al., 2003; Ivanoff et al., 2008; Odoemene et al., 2018).

In summary, this study first argues theoretically, using the reflective-impulsive model, that two types of processing take place in message framing that explain how the different effects of message framing and emotional responses come about. At the neural level, using the network perspective, it is assumed that some brain regions are primarily associated with emotion and cognition interaction processes. The results show that these regions in particular are also active during processing, depending on the message framing. This could be a first indicator for the two-process logic and supports a legitimate application of the model since it has an inherent dual character of interacting processes. The study shows that an experiential “gut feeling” awareness might play a major role; however, this effect is not reflected in a change in behavior. By theorizing and integrating neural perspectives, this study can help people to better understand this “gut feeling,” and especially help scientists to describe it and form hypotheses from it, because they become able to express this phenomenon in theoretical terms. The reflective-impulsive model potentially provides a toolkit with which to examine these experiential bodily sensations, as some of the components from which these feelings results are known.

### 6.1. Limitations

Overall, the results provide first support for an interpretation of message framing effects according to a reflective-impulsive model. However, as with any study, there are some limitations that should be noted.

First, again special attention must be paid to the problem of backward interference (Poldrack, 2011; Plassmann et al., 2015; Glymour and Hanson, 2016). Accordingly, it cannot be assumed that a certain processing type is operating when a specific brain area is activated. However, the neural activations associated with message framing processing appear to correlate with the activation of brain areas associated with the integration of emotion-cognition interactions. The results thus provide first insights that message framing processing involves brain areas that are correlated with similar processing styles as assumed in the theoretical model of message framing. Still, it cannot be assumed that the brain areas indicate the impulsive or reflective process. The activations indicate a potential interaction between affective and cognitive processes, assuming a network perspective of the brain (Pessoa, 2012, 2017).

Second, when interpreting the results, some methodological conditions must be taken into account, which limit the generalizability. The sample of the fMRI study included only right-handed people. Although this is a common practice in neuroscience research, an ongoing discussion about this exclusion criterion raises questions about the generalizability of the results (Bailey et al., 2020). Furthermore, only participants who consume meat products were included, limiting the sample to people whose consumption behavior is directly affected by products from livestock farming and who may be less morally critical of the chosen topic (Bennett et al., 2002; de Jonge et al., 2015). As the brain structures involved are highly influenced by biological factors, such as their decline with age, and their capacity depends on mental load (MacPherson et al., 2002; Sowell et al., 2003; Pardo et al., 2007), other external factors could also be tested in relation to message framing (e.g., age). This would allow the theoretical model to be validated for other populations.

Third, previous literature has identified different message framing dimensions that can be manipulated by the frame (positive-negative, concrete-abstract, self-other; Florence et al., 2022). While this study focuses primarily on the positive-negative value dimension, future studies could expand the stimulus sets and investigate different combinations of message dimensions. The hypothesized effects should then in turn be interpreted against the background of the reflective-impulsive model to see whether clear behavioral and neural effects can be derived and tested in these cases as well.

### 6.2. Implications

Based on these findings, implications for theory and practice can be derived.

For practice, the results show that different communication strategies should be used depending on object value. While positive frames should be used for positive object values, negative frames seem to be only a persuasive communication strategy for negative object values. In this case, any additional explanation is favorable as it seems to increase the immediate evaluation of the object. Moreover, according to the theoretical interpretation of the reflective-impulsive model, negative frames might be even more advisable as they could create a positive, supportive experiential responsiveness to the message framing. In the case of negative object value, it could be more favorable to also communicate the “bad,” negative consequences in the appropriate frame. Since there are indications of the neural mechanisms involved in message framing, this could also be a starting point for future research to relate socio-demographic variables to it. This could reveal differences in the effects of message framing and, as a result, perhaps targeting people differently in terms of the types of framing.

Theoretically, the integration of neuroscience contributes to explain contradictory effects and inconclusive results in research on message framing. This should bring a new perspective to the discussion, from which one can then perhaps see how the differing effects of message framing might originate. By adopting different approaches to the study of affective and cognitive mechanisms and integrating them into a theoretical explanatory model, different effects can be interpreted at the individual level and their implications extended to the level of society, as well as within a variety of organizations. However, the question of whether such a theoretical model can be validated in other studies of cognitive and neurophysiological mechanisms that influence behavior on a large scale needs to be explored in future research.

## Data availability statement

The datasets presented in this article are not readily available because it was ensured to the participants that their non-anonymous data is not available for third parties, and it was guaranteed that participants can request the complete deletion of their non-anonymous datasets at any time. Requests to access the datasets should be directed to nadine.gier@hhu.de.

## Ethics statement

The studies involving humans were approved by Ethik-Kommission—Medizinische Fakultät Bonn, Biomedizinisches Zentrum, Rheinische Friedrich-Wilhelms-Universität: ethics number (390/16). The studies were conducted in accordance with the local legislation and institutional requirements. The participants provided their written informed consent to participate in this study.

## Author contributions

NG organized the conceptualization of the study, supervised data acquisition, performed preparation, analysis and interpretation of the results, and wrote the first draft of the manuscript. CK and PK contributed to conception and design of the study. All authors contributed to manuscript revision, read, and approved the submitted version.
